# A New Call for Nurses

**Published:** 1920-09-18

**Authors:** 


### A New Call for Nurses.

The Ministry of Health has issued its first annual report of the department dealing with tuberculosis for 1919-20. The details given open up an astonishingly large field of work, in every branch of which nurses are an indispensable factor. Up to the end of last March nearly 400 dispensaries for the treatment of tuberculosis had been approved, entailing the employment of a staff of whole-time visiting nurses wherever the organisation is complete. The number of residential institutions is now 388, and the number of beds, increased during the year by the addition of 2,258, totals 15,781. Proposals for providing nearly 10,000 additional beds have been submitted. This means a great army of nurses trained in a special manner. It must never be forgotten that the nature of the disease makes it imperative for all who are engaged in combating it to lead a rigorously healthy life. The eight-hour day, the weekly day's leave, are imperative in this branch of the nursing service. We wish we could add that they were universal. It is satisfactory to learn that the preventive measures now in force are taking effect. As compared with last year, notifications have been reduced by 11,902 in the case of pulmonary, and 2,440 in the case of other forms of tuberculosis. Deaths have decreased by 9,415 and 2,346 respectively.

